# Targeting DNA Double-Strand Break Repair Pathways to Improve Radiotherapy Response

**DOI:** 10.3390/genes10010025

**Published:** 2019-01-04

**Authors:** Mahmoud Toulany

**Affiliations:** Division of Radiobiology and Molecular Environmental Research, Department of Radiation Oncology, University of Tuebingen, Roentgenweg 11, 72076 Tuebingen, Germany; mahmoud.toulany@uni-tuebingen.de; Tel.: +49-(0)7071-298-5832; Fax: +49-(0)7071-295-900

**Keywords:** double-strand break repair, ionizing radiation, signal transduction, molecular targeting, radiosensitization

## Abstract

More than half of cancer patients receive radiotherapy as a part of their cancer treatment. DNA double-strand breaks (DSBs) are considered as the most lethal form of DNA damage and a primary cause of cell death and are induced by ionizing radiation (IR) during radiotherapy. Many malignant cells carry multiple genetic and epigenetic aberrations that may interfere with essential DSB repair pathways. Additionally, exposure to IR induces the activation of a multicomponent signal transduction network known as DNA damage response (DDR). DDR initiates cell cycle checkpoints and induces DSB repair in the nucleus by non-homologous end joining (NHEJ) or homologous recombination (HR). The canonical DSB repair pathways function in both normal and tumor cells. Thus, normal-tissue toxicity may limit the targeting of the components of these two pathways as a therapeutic approach in combination with radiotherapy. The DSB repair pathways are also stimulated through cytoplasmic signaling pathways. These signaling cascades are often upregulated in tumor cells harboring mutations or the overexpression of certain cellular oncogenes, e.g., receptor tyrosine kinases, *PIK3CA* and *RAS*. Targeting such cytoplasmic signaling pathways seems to be a more specific approach to blocking DSB repair in tumor cells. In this review, a brief overview of cytoplasmic signaling pathways that have been reported to stimulate DSB repair is provided. The state of the art of targeting these pathways will be discussed. A greater understanding of the underlying signaling pathways involved in DSB repair may provide valuable insights that will help to design new strategies to improve treatment outcomes in combination with radiotherapy.

## 1. DNA Double-Strand Break Repair

Despite advances in radiotherapy, radioresistance remains a major cause of treatment failure, leading to lower progression-free survival rates in cancers such as lung cancer, pancreatic cancer, and glioblastoma. This failure indicates the necessity to investigate the underlying network of signal transduction pathways known as DNA damage response (DDR) pathways, which stimulate the repair of double-strand breaks (DSBs), the most lethal type of DNA damage due to radiotherapy [1]. Following exposure to ionizing radiation (IR), cells undergo transient cell cycle arrest to perform DNA repair. DSBs are repaired either by classical and alternative non-homologous end joining (C-NHEJ and A-NHEJ) throughout the cell cycle or by homologous recombination (HR) during the S phase and G2 phase [2]. After the induction of DSB, the MRE11-Rad50-NBS1 (MRN) complex and K70/80 act as sensor proteins to recognize the lesions [3,4]. Thereafter, the *phosphoinositide* 3-kinase (*PI3K*) family members ataxia telangiectasia mutated (ATM), ataxia telangiectasia and Rad3-related (ATR), and DNA-dependent protein kinase catalytic subunit (DNA-PKcs) are activated [5]. The activation of ATM kinase by autophosphorylation relays the signal to the transducer enzymes, including checkpoint kinase 2 (CHK2) and the transcription factor p53. Cellular p53 regulates the expression of cell cycle regulators such as p21 that, through interaction with the cyclin-dependent kinase (CDK) complex, lead to G1 arrest. Along with this process, chromatin modification occurs, and DNA repair is initiated [6]. In most cancer cells, the G1 checkpoint is defective because of mutations in the key regulators of the G1 checkpoint, such as p53. Therefore, the G2 checkpoint becomes important for cancer cell survival. ATR phosphorylates checkpoint kinase 1 (CHK1). The activation of CHK1 mediates the phosphorylation of cell division cycle 25A (CDC25A) and consequently its degradation, which slows the progression of DNA replication through the S phase. Likewise, CHK1 phosphorylates CDC25C, which stops the cell cycle in the G2 phase [7,8]. After arresting in different cell cycles, cells apply the canonical DSB repair pathways to repair DSBs before cell cycle re-entry. Thus, the underlying components involved in cell cycle arrest and canonical DSB repair pathways can be targeted to block DSB repair and induce radiosensitivity. More details on the canonical DSB repair pathways have been presented in review articles by other investigators [4,9,10,11].

Tumor-specific defects in DDR provide new options for cancer therapy. A significant fraction of cancers have inherited and acquired defects in one of the DNA repair pathways [5,12]. One example is the defect in the HR repair pathway caused by mutations in the tumor suppressor genes *BRCA1* and *BRCA2*, which are related to breast and ovarian cancer. Mutation in the *p53* gene, which is referred to as the guardian of the genome and is most commonly mutated in human cancers, is another example of tumor-specific DDR defects. DNA damage repair in cancer cells that have lost one DNA repair component usually requires other repair pathways to allow the cells to survive after therapy. Thus, targeting these rescue repair pathways in cancer cells with repair deficiency, known as synthetic lethality, might be an effective therapeutic approach for such cancers [13]. Synthetic lethality is the underlying mechanism by which BRCA1/2-defective tumor cells are sensitive to poly(ADP-ribose) polymerase (PARP) inhibitors. Likewise, in *p53*-mutated cells with defects in the G1/S checkpoint, targeting the G2/M checkpoint becomes a very effective therapy to sensitize *p53* mutant cells to DNA damage-inducing agents such as ionizing radiation (IR) during radiotherapy. Application of the concept of synthetic lethality to radiotherapy may be an effective approach to enhance the therapeutic ratio by minimizing normal-tissue side effects [14].

**Figure 1:**
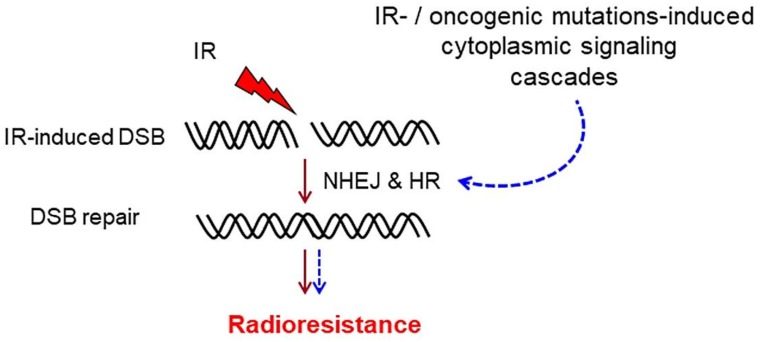
Cytoplasmic signaling cascades mediate post-irradiation cell survival and radioresistance in tumor cells by stimulating the canonical DSB repair pathways in the nucleus. Downstream effectors of cytoplasmic signaling cascades translocate to the nucleus and stimulate the canonical DSB repair pathway through either direct physical interaction with the repair protein or indirect stimulation of the pathway. IR: ionizing radiation; DSB: double-strand break; NHEJ: non-homologous end joining; HR: homologous recombination.

Exposure to IR induces the activation of cytoplasmic signaling cascades, such as the PI3K/AKT, RAS-mitogen-activated protein kinase (MAPK), signal transducer and activator of transcription (STAT) and phospholipase *C* (PLC) pathways [15,16]. According to the functions of such pathways, the radiation-induced activation of these signaling cascades accelerates cell proliferation and the post-irradiation survival of tumor cells [17,18,19,20]. In addition to the well-described role of the cytoplasmic signaling pathway in regulating multiple cellular functions, the activation of these pathways stimulates the repair of radiation-induced DSBs by regulating the expression and/or activation of the components of the canonical DSB repair pathways. It is notable that the described functions of these pathways in DSB repair and radioresistance are not solely dependent on IR-induced stimulation of the underlying pathways. These cascades can also become hyperactivated in tumor cells expressing mutations or overexpressing certain oncogenes or tumor suppressor genes, elevating the DSB repair capacity of these cells. Thus, as depicted in Figure 1, this mode of action in DSB repair may specifically lead to the radioresistance of tumor cells. Likewise, it provides a rationale for mutational screening and expression analyses of previously described oncogenes before radiotherapy. The knowledge obtained from such analyses will help to design appropriate molecular therapy strategies in combination with radiotherapy as part of personalized therapy.

In the present paper, the relevant literature concerning the activation of canonical DSB repair pathways through cytoplasmic signaling cascades is summarized. The information regarding the described signaling cascades is accompanied by a description of proof-of-principle radiosensitization by targeting the components of the described pathway.

## 2. Receptor Tyrosine Kinases That Mediate DSB Repair after Irradiation

Membrane-bound receptor tyrosine kinases (RTKs) consist of 58 members within 20 subfamilies. Mutation or overexpression of these receptors has been reported in more than 65% of these receptor families in different cancers. These alterations result in deregulated kinase activity and malignant transformation [21] and are associated with poor prognosis, drug resistance, cancer metastasis, and lower survival rate. In addition to the contributions of direct genomic events to the expression and hyperactivation of RTKs, epigenetic modifications of RTKs also modulate RTK activity [22,23].

### 2.1. ErbB Family of RTKs

**Figure 2:**
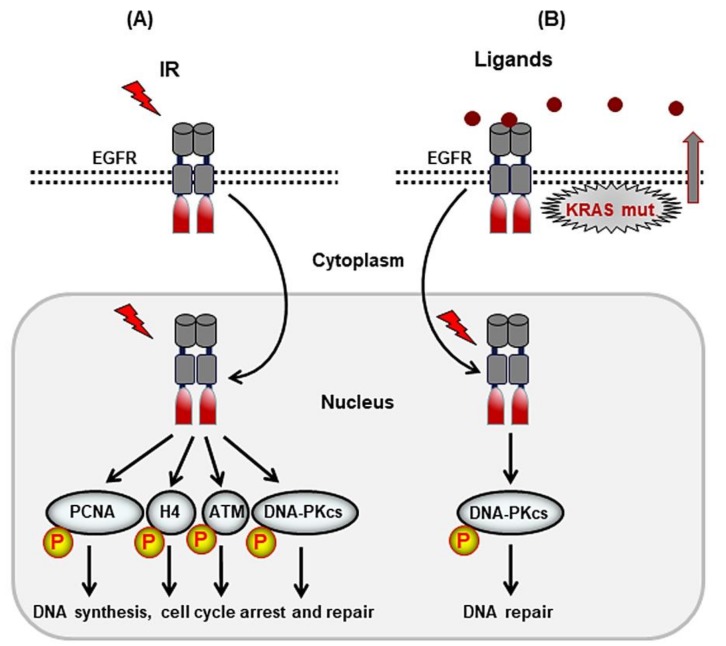
Overview of potential mechanisms by which epidermal growth factor (EGFR) stimulates DNA double-strand break repair, leading to radioresistance. (**A**) Ionizing radiation (IR) stimulates membrane-bound EGFR, which may lead to translocation of the receptor to the nucleus. Ionizing radiation may induce the activation of EGFR directly in the nucleus. (**B**) Exogenous stimulation of EGFR by related ligands, such as EGF and transforming growth factor α (TGFα), as well as autocrine secretion of EGFR ligands, such as in tumor cells harboring the *KRAS* mutation, induce nuclear translocation of EGFR. Following irradiation, activated EGFR in the nucleus stimulates DNA repair machinery by stimulating the phosphorylation/activation of proteins involved in DNA damage response (DDR) and DSB repair. EGFR may also function as a transcription factor regulating proteins involved in DSB repair.

The ErbB receptor family, also described as the epidermal growth factor (EGFR) family, includes EGFR or ErbB1/Her1, ErbB2/Her2, ErbB3/Her3, and ErbB4/Her4. These family members are activated after homodimerization or heterodimerization. No ligand has been described for ErbB2, and ErbB3 has impaired kinase activity. Among the RTKs, nuclear localization of the ErbB receptor family members EGFR, HER2, and HER3 has been reported. The first step in the function of RTKs in DSB repair is the nuclear localization of the receptors. Potential mechanisms involved in the nuclear translocation of these receptors, including endosome-mediated nuclear translocation and retro-translocation by the endoplasmic reticulum, have been shown and reviewed elsewhere [24]. RTKs stimulate DNA repair through several mechanisms. In this context, a critical role of EGFR acetylation has been reported to regulate tyrosine phosphorylation of the receptor and its function [23]. Likewise, nuclear EGFR can directly interact with DSB repair proteins such as DNA-PKcs, a core enzyme in the NHEJ repair pathway that stimulates DSB repair after irradiation [25,26,27]. Radiation may also stimulate EGFR activity in the nucleus without requiring translocation from the cytoplasm to the nucleus, although this issue has not yet been investigated (Figure 2A). Stimulating cells with EGFR ligands, e.g., EGF and transforming growth factor α (TGFα), leads to the translocation of EGFR to the nucleus [28]. The expression of oncogenic *KRAS* stimulates autocrine secretion of TGFα and another EGFR ligand, amphiregulin [29,30,31]. Because the overexpression of oncogenic *KRAS* leads to enhanced radiation-induced autophosphorylation of DNA-PKcs at S2056 and stimulates DSB repair [29], it is supposed that the translocation of activated EGFR to the nucleus facilitates radiation-induced DNA-PKcs activity and stimulates DSB repair through NHEJ. According to the model outlined in Figure 2B, nuclear EGFR was proposed to play an efficient role in DSB repair through NHEJ in tumor cells expressing oncogenic *KRAS*.

EGFR also plays a key role in the repair of DSBs through HR. Nowsheen et al. showed that EGFR, in a protein complex with BRCA1, plays an essential role in DSB repair through HR [32]. Lee et al. showed that, upon irradiation, ATM associates with and is phosphorylated by EGFR at Tyr-370 at the site of DSBs. Likewise, EGFR regulates the phosphorylation of ATM at Tyr-370, leading to Chk2 activity [33], which can affect G1 cell cycle arrest, a prerequisite for NHEJ. Additionally, Wang and colleagues showed that chromatin-bound proliferating cell nuclear antigen (PCNA) phosphorylation at Tyr-211 is dependent on the tyrosine kinase activity of EGFR in the nucleus. Phosphorylation at Tyr-211 by EGFR stabilizes the chromatin-bound PCNA protein and its associated functions [34]. EGFR also mediates the Tyr-72 phosphorylation of histone 4 (H4), which leads to the Lys-20 methylation of H4 and the acceleration of DNA synthesis and repair [35]. In the context of epigenetic regulation by RTKs, it was also reported that EGFR acetylation plays a role in the function of the receptor, leading to tumor cell resistance to histone deacetylase inhibitors [23]. These and other lines of evidence strongly indicate that EGFR regulates DDR and stimulates DSB repair through both NHEJ and HR, as summarized in Figure 2. Consistent with the role of EGFR in regulating the DDR response, i.e., stimulating DSB repair through HR and NHEJ [36,37,38,39], EGFR expression is inversely correlated with the IR sensitivity [40] and overall survival of patients after radiotherapy [41].

### 2.2. Targeting the ErbB Family of RTKs in Combination with Radiotherapy

The ErbB family of receptors has been considered the most important target in oncology [42,43] as well as in radiation oncology [44]. Among the molecular targeting approaches, inhibitors against EGFR, i.e., monoclonal antibodies and tyrosine kinase inhibitors (TKIs), have been thoroughly investigated in several preclinical and clinical settings, as reviewed by Huang et al. [45]. As a proof of concept, the anti-EGFR antibody cetuximab has been shown to improve radiation sensitivity in head and neck squamous cell carcinoma (HNSCC) in multicenter phase III clinical trials [46,47]. To date, the majority of studies performed with anti-EGFR antibodies have been conducted using cetuximab. Most of these clinical trials have been performed in HNSCC patients in combination with chemoradiotherapy. However, as summarized in Table 1, only a single phase III trial for the combination of cetuximab with radiotherapy led to improved overall survival in HNSCC patients [46,47]. The combination of cetuximab with chemoradiotherapy was not positive and sometimes even showed inferior outcome (Table 1) The combination of cetuximab with chemoradiotherapy for other tumor entities was optimized according to the aim of each study [48,49,50]. However, treatment-associated toxicity is often a limiting factor that must be considered [51,52]. The beneficial impact of EGFR TKIs has been mainly investigated by the combination of erlotinib and gefitinib with radiotherapy or chemoradiotherapy in non-small cell lung cancer (NSCLC) as well as HNSCC. Some of the early phase clinical trials combining erlotinib and radiotherapy or chemoradiotherapy are summarized and presented in Table 1.

Radiation-induced DSB is the major mechanism by which radiotherapy induces cell death. Thus, interference with the DSB repair pathway may be the crucial mechanism by which cetuximab improves radiotherapy outcomes. Additionally, EGFR targeting enhances IR-induced apoptosis, inhibits proliferative growth, and downregulates tumor angiogenic response [63].

Although the nuclear localization of Her2 and Her3 has been demonstrated previously [64,65,66], the specific function of these receptors in DSB repair is largely unknown. Consistent with the role of nuclear Her3 in radioresistance [67], Reif et al. showed that the level of nuclear Her3 increases upon hypoxia [66]. Because hypoxia is associated with radioresistance, it may be concluded that the enhanced nuclear translocation of Her3 is linked to DSB repair.

### 2.3. IGF-1 Receptor

The membrane-bound type 1 insulin growth factor receptor (IGF-1R) is a tyrosine kinase receptor that is stimulated by IGF-1, a polypeptide protein hormone similar in molecular structure to insulin. IGF-1R signaling contributes to the growth of many solid tumors, such as glioblastoma [68], with poor prognosis. IGF-1R is the second most studied receptor whose roles in DSB repair and radioresistance have been reported. Nuclear localization of IGF-1R has been well demonstrated by several investigators [69,70] and is associated with therapy resistance. Likewise, high levels of total or cytoplasmic IGF-1R expression reveal an increased risk of postradiotherapy recurrence in prostate cancer patients [71], indicating that radioresistance is mediated by IGF-1R.

### 2.4. Targeting IGF-1R in Combination with Radiotherapy

Because suppression of HR sensitizes human tumor cells to IGF-1R inhibition, IGF-1R might be crucial for HR repair of DSBs [72]. In addition to its role in HR, IGF-1R also stimulates NHEJ repair of DSB [73,74]. These results and other evidence of the role of IGF-1R in DSB repair support the idea that this receptor can potentially be an appropriate target in combination with radiotherapy. To date, several studies combining IGF-1R targeting with IR have been reported. Similar to the EGFR targeting strategies, monoclonal antibodies and RTKs have been investigated as potential approaches to block IGF-1R in combination with radiotherapy. The anti-IGF-1R monoclonal antibodies A12 (ImClone Systems, Inc., New York, NY, USA) and CP-751,871 enhance the radiosensitivity of NSCLC via the inhibition of DSB repair [75,76]. In addition to the monoclonal antibodies, the radiosensitizing effect of IGF-1R TKIs has also been investigated. Isebaert et al. demonstrated that the IGF-1R TKI NVP-AEW541 enhances radiosensitivity in phosphatase and tensin homolog (PTEN) wild-type but not PTEN-deficient human prostate cancer cells [77]. NVP-AEW541-induced radiosensitization was associated with downregulation of phospho-AKT levels and high levels of residual DSB [77], indicating a role for IGF-1R in stimulating DSB repair pathways. Chitnis et al. demonstrated that the radiosensitizing effect of the IGF-1R TKI AZ12253801 depends on the expression of DNA-PKcs [73]. AZ12253801 induced radiosensitization in DNA-PKcs-proficient but not DNA-PKcs-deficient glioblastoma cells and did not radiosensitize DNA-PKcs-inhibited DU145 prostate cells [73]. These results suggest that IGF-1R functions in the same pathway as DNA-PKcs, which stimulates the C-NHEJ repair of DSB repair [73]. The radiosensitizing effect of IGF-1R molecular targeting strategies has also been supported by genetic approaches. Zhao and Gu reported that the silencing of IGF-1R enhances the radiation sensitivity of human esophageal squamous cell carcinoma, leading to tumor growth delay and prolonged survival after radiotherapy in a tumor xenograft model in vivo [78]. IGF-1R has a functional interaction with EGFR [79]. This interaction stimulates the activation of common downstream signaling cascades, e.g., the PI3K/AKT pathway. Consistent with this conclusion, investigation of co-targeting IGF-1R and EGFR in gastric cancers has been suggested [80] and might be an efficient approach to improve radiotherapy outcome. Interference with the PI3K/AKT pathway is one of the potential mechanisms by which targeting IGF-1R induces radiosensitization [77]. However, because the PI3K/AKT pathway is a common pathway downstream of all RTK members, including EGFR, the long-term inhibition of IGF-1R may lead to the activation of this pathway through EGFR. This compensatory reactivation of the PI3K/AKT pathway may diminish the radiosensitizing effect achieved by targeting IGF-1R.

### 2.5. TAM Family of Receptors

The TAM family of RTKs consists of three members: TYRO-3, AXL, and MERTK. TAM receptors play an important role in promoting the growth, survival, and metastatic spread of several tumor types. AXL and MERTK have been described to be overexpressed in HNSCC, triple-negative breast cancer (TNBC), and NSCLC, malignancies that are highly metastatic and lethal [81]. The level of TAM receptor expression correlates with the tumor grade and emergence of chemo- and radioresistance to targeted therapeutics [82]. The AXL receptor and its activating ligand, growth arrest-specific 6 (GAS6), are important drivers of metastasis and therapeutic resistance [83,84]. Thus, based on the stimulatory role of AXL in DNA repair and therapy resistance, targeting AXL might be an effective approach to improve radiotherapy outcome.

### 2.6. Targeting AXL in Combination with Radiotherapy

The downregulation or inhibition of AXL decreases the expression of DNA repair genes and diminishes the efficiency of HR [85]. Consistent with the function of AXL in DSB repair, the expression of AXL is highly associated with the radiation resistance of human papilloma virus (HPV)-negative HNSCC cells, dependent on the activation of PI3K and the expression of programmed death-ligand 1 (PD-L1) [86]. Based on the role of AXL in DNA repair and radioresistance [85,86], targeting AXL is supposed to be an efficient approach to block DSB repair and induce radiosensitization. An anti-AXL antibody and small molecule TKIs of AXL have been investigated in preclinical trials, and some have entered clinical trials. The antitumor effectiveness of the AXL small-molecule inhibitors TP-0903 [87] and R428 [88] has been tested in hematological malignancies and solid tumors [87,89,90]. However, cancer treatment by targeting AXL is a relatively new field in oncology, and thus far, few investigations have been conducted in combination with radiotherapy. In this context, Brand et al. demonstrated that the AXL inhibitor R428 blocks DSB repair and induces radiosensitivity [91]. Additionally, because AXL mediates the nuclear localization of EGFR and resistance to anti-EGFR therapy [92], co-targeting of AXL and EGFR might be an efficient approach to induce radiosensitization, a strategy that needs to be investigated.

## 3. Cytoplasmic Signaling Cascades That Stimulate DSB Repair

In addition to the direct function of RTKs in DSB repair as described above, this family of receptors can also stimulate the repair of DSB by activating various cytoplasmic signaling pathways, such as the PI3K/AKT pathway, STAT pathway, and RAS-MAPK pathway. Among the different pathways downstream of RTKs, the PI3K/AKT pathway is the best-studied pathway regulating DNA damage repair. This pathway is one of the major survival pathways and is frequently upregulated in tumors from different entities [93,94,95,96].

### 3.1. Stimulation of DSB Repair by the PI3K/AKT Pathway

Mutations in *RTKs*, *PTEN*, *PI3K*, *AKT*, and *RAS* are the major mutations involved in the constitutive activation of the PI3K/AKT pathway. Additionally, exposure to IR induces the activation of this pathway through the stimulation of upstream RTKs, e.g., EGFR [17,97,98,99,100,101]. Targeting PI3K induces radiosensitization in tumor cells from different entities in vitro and in vivo [17,97,100,102,103,104,105,106]. Initial studies using the PI3K inhibitor LY294002 showed a radiosensitizing effect through interference with DSB repair pathways, i.e., HR and NHEJ [107,108,109].

Among the three classes of PI3K, the class IA isoform of PI3K has been strongly implicated in cancer. This PI3K isoform consists of a p85 regulatory subunit and a p110 catalytic subunit. Despite the therapeutic benefit of targeting this isoform, there have been concerns about the severe adverse effects of this class of drug that have led to difficulties in the application of PI3K inhibitors in combination with radiotherapy [110]. PI3K activity regulates various substrates that are involved in different cellular functions, such as cell cycle progression, different types of cell death, glycolysis, and DNA repair [93,111]. Among the many PI3K substrates, AKT, also known as protein kinase B (PKB), is the key mediator of the PI3K signaling pathway [112]. AKT is involved in the repair of radiation-induced DSBs through both HR and NHEJ [99,107,109,113,114,115,116,117]. Consistent with the preclinical observations, the clinical data support the role of AKT in radioresistance. In this context, AKT activity was reported to be a prognostic marker for the radiotherapy response of head and neck cancer and cervical cancer patients [118,119]. The PI3K-independent reactivation of AKT has been reported in *KRAS*-mutated NSCLC cells [120,121,122], glioblastoma cells [123], bladder cancer cells [124], and TNBC cells [125] after the blockade of PI3K. Thus, in addition to dose-limiting toxicities, the feedback activation of AKT can be an obstacle for targeting PI3K and limits the success of combining PI3K inhibitors with radiotherapy.

AKT consists of three isoforms—AKT1/PKBα, AKT2/PKBβ, and AKT3/PKBγ —transcribed from separate genes. These AKT isoforms have an N-terminal pleckstrin homology (PH) domain and a kinase domain, which are separated by 39 amino acids [126]. The PH domains and kinase domains in the AKT isoforms are approximately 60% and 85% identical, respectively [127]. Activated AKT regulates the function of numerous substrates in cell growth, proliferation, and survival, as well as regulating metabolism, angiogenesis, and migration [128]. Among the different AKT isoforms, AKT1 was the first isoform that was described to directly interact with DNA-PKcs [116,129] through its C-terminal domain [116]. Furthermore, we showed that, similar to AKT1, AKT3 also interacts with DNA-PKcs [130]. No interaction with DNA-PKcs could be observed for AKT2 in *KRAS*-mutated NSCLC [130]. Thus, AKT activates DNA-PKcs kinase activity and its phosphorylation at Thr-2609 and Ser-2056, which is essential for DSB repair by NHEJ [131,132]. Activated AKT localizes to DSB sites, as shown by the colocalization of γH2AX foci as a marker of DSB with P-AKT (Ser-473) after irradiation [115,133,134], and stimulates DSB repair, leading to radioresistance [116,130,135,136,137,138]. Accumulating evidence has indicated the role of AKT in stimulating HR. My colleagues and I recently showed that AKT1 knockdown reduces Rad51 protein level, Rad51 foci formation, and its colocalization with H2AX foci after irradiation [117]. These events were associated with unsuccessful HR, as shown by increased BRCA1 foci 24 h post-irradiation [117]. Consistent with the role of AKT in HR, it was shown that, in *PTEN*-mutated cells with the hyperactivation of PI3K, PI3K inhibition reduces RAD51 foci formation and sensitizes these cells to the PARP inhibitor [139].

**Figure 3:**
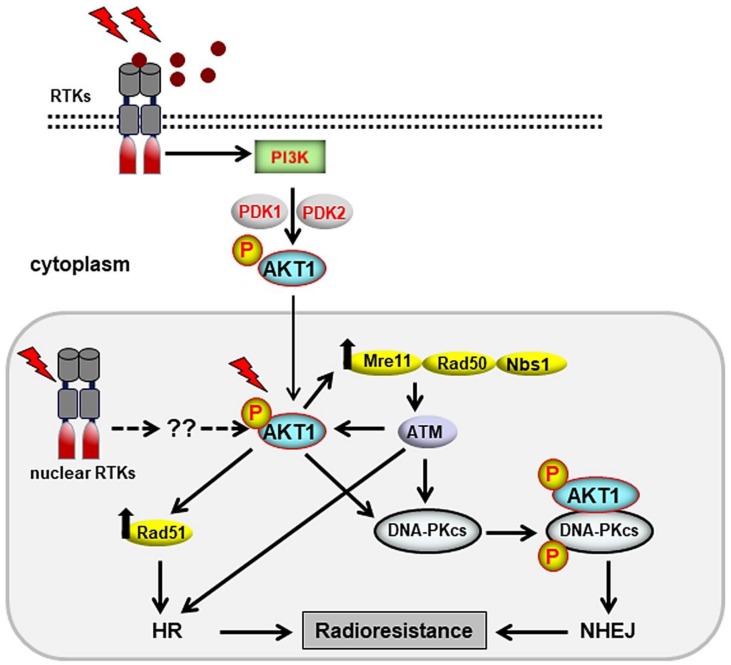
AKT1 stimulates DSB repair through the NHEJ and HR repair pathways. Exposure to IR induces the activation of cytoplasmic AKT1 that may translocate to the nucleus. Alternatively, IR can activate AKT1 by stimulating nuclear receptor tyrosine kinases (RTKs) independent of cytoplasmic AKT1. Activated AKT1 in the nucleus stimulates the DNA repair machinery by increasing the expression of repair proteins such as Mre11 and Rad51 or inducing the phosphorylation/activation of proteins involved in DDR and DSB repair.

AKT, through the GSK3β/β-catenin/LEF pathway, upregulates the expression of Mre11 in the MRN complex and elevates DSB repair capacity [140]. The MRN complex recruits ATM to the DSB sites, where ATM is subsequently activated. Activated ATM, similar to phosphorylating members of the MRN complex, stimulates the phosphorylation of AKT at Ser-473 [141] through RNF168 [115]. Given the role of AKT in MRE11 expression [140], AKT leads to the expression of MRE11 and stimulation of ATM signaling. Because ATM plays a critical role in regulating HR repair [142], AKT stimulates HR as well. Thus, the ATM-dependent mode of action of AKT in HR might be important to regulate the slow component of DNA repair following the fast NHEJ repair process. AKT in non-irradiated cells is expressed in the nucleus. Thus far, there is no convincing data for IR-induced nuclear translocation of AKT. Because AKT needs to be in the nucleus immediately after irradiation to play its role in regulating DNA repair pathways, it can be assumed that nuclear AKT is activated by PI3K components in the nucleus, independent of the cytoplasmic fraction, although this issue needs to be investigated. Overall, the role of AKT in the stimulation of DSB repair through HR as well as NHEJ makes AKT an effective target in combination with radiotherapy. Some of the most important aspects of the function of AKT in DSB repair are summarized in Figure 3.

The hyperactivation of AKT correlates with various clinicopathological parameters and is a prognostic indicator for cancers from different entities [143,144,145,146]. The mutation and overexpression of oncogenes such as RTK families, *RAS*, and *PIK3CA*, as well as mutation in the tumor suppressor gene *PTEN*, lead to stimulated AKT activity. The constitutive activation of KRAS through point mutation in *KRAS* gene leads to the activation of the PI3K/AKT and MAPK/ERK pathways, as the major pathways regulating growth proliferation and survival. It is well known that mutation in the *KRAS* gene leads to radioresistance that is linked to the activation of the PI3K/AKT pathway [29,147]. Thus, it is supposed that KRAS or PI3K may serve as a suitable target to overcome radioresistance. To this end, several laboratories have begun testing the antitumor activity of KRAS mutation-specific inhibitors. Ostrem et al. validated a new allosteric regulatory site on KRAS (G12C) that is targetable in a mutant-specific manner, and the described inhibitor blocks the interaction of KRAS protein with effectors such as B-Raf and C-Raf [148]. However, in that study, the impact of KRAS (G12C) inhibition on PI3K/AKT activity was not investigated. In a further study, Misale et al. [149] demonstrated that the reactivation of the PI3K/AKT pathway limits the efficacy of the KRAS (G12C) inhibitor ARS1620 in some of the cell lines tested. These authors suggested the combination of ARS1620 with PI3K inhibitors [149]. This resistance mechanism indicates the rationale for targeting the PI3K/AKT survival pathway alone as well as in combination with IR. Previously, we showed that short-term treatment (2 h) with the PI3K inhibitor PI-103 blocks the phosphorylation of AKT (Ser-473 and Thr-308) as well as the phosphorylation of the AKT substrate PRAS40 (Thr-246) in NSCLC cell lines A549 and H460 expressing *KRAS* (G12V) and *KRAS* (Q61H), respectively. The inhibitory effect of PI-103 disappeared at 24 h post-treatment in both *KRAS*-mutated cells [121]. In *KRAS* wild-type H661 cells, PI-103 blocked AKT phosphorylation at the 2-h and 24-h post-treatment time points [120,121]. These new observations and previous reports on the role of oncoprotein RAS, especially KRAS, in activating survival pathways warrant further studies to uncover appropriate targeting strategies to overcome RAS-mediated radioresistance.

Among the three AKT isoforms tested, AKT1 and AKT3 interact with DNA-PKcs and regulate DSB repair through NHEJ [130]. Mutations in *AKT* isoforms lead to the constitutive activation of AKT. The E17K mutation in the *AKT1* gene causes the constitutive membrane localization of AKT1. This results in permanent AKT1 kinase activity and phosphorylation at Thr-308 and Ser-473, as well as the consequent activation of downstream target proteins independent of growth factor stimulation, as reported in different cancers such as breast cancer, endometrial cancer, bladder cancer, lung cancer, and colorectal cancer [150,151,152,153]. The expression of AKT1-E17K accelerates DSB repair and improves post-irradiation cell survival [154]. For full activity, AKT3 needs to be phosphorylated at Ser-472 and Thr-305 [127]. Similar to the *AKT1* mutation, the E17K mutation in the *AKT3* gene leads to the constitutive activation of AKT3.

### 3.2. Targeting AKT for Radiosensitization

Based on the role of AKT in DSB repair discussed above and the common disruption of the RTK/PI3K/AKT pathway in human cancers, e.g., the hyperactivation of AKT in over 50% of human tumors, AKT is a suitable target in combination with radiotherapy. Thus far, almost all the relevant studies, particularly clinical trials, have been performed using AKT inhibitors that inhibit all three isoforms. The targeting of AKT can be achieved using either allosteric inhibitors or ATP competitive inhibitors. Allosteric inhibitors prevent the plasma membrane localization of AKT through blocking the PH domain. Thus, AKT will not be recruited to the cell membrane, where it is phosphorylated at its threonine and serine residues by PDK1 and PDK2, respectively. Several small-molecule AKT inhibitors are currently undergoing clinical evaluation. The current status of clinical trials with AKT inhibitors as monotherapy or in combination with chemotherapy has been summarized by other investigators [155].

Preclinical data support the radiosensitizing effect of AKT inhibitors through blocking DSB repair [136,156,157]. Among several AKT inhibitors, phase I trials have been performed using the combination of the AKT inhibitor perifosine with radiotherapy. Phase I and pharmacokinetic studies of combined treatment with perifosine and radiation in patients with advanced solid tumors have indicated that perifosine can be safely combined with fractionated radiotherapy [158]. Nelfinavir is a human immunodeficiency virus (HIV) protease inhibitor that has been shown to possess antitumor activity and radiosensitizing effects through the inhibition of phosphorylated AKT [159]. The combination of nelfinavir and chemoradiotherapy in phase I and II clinical trials showed acceptable toxicity and promising activity in patients with NSCLC, rectal cancers, and pancreatic cancers [160,161,162,163,164] (Table 2), which frequently express *KRAS* mutations. Overall, thus far, mechanistic in vitro studies and existing clinical trials have supported the rationale for future, more in-depth clinical studies investigating the combination of AKT inhibitors with radiotherapy.

## 4. Conclusions and Prospects

Cytoplasmic signaling cascades downstream of oncoproteins, such as RTKs and RAS, stimulate canonical DSB repair pathways, i.e., NHEJ and HR. Direct targeting of the canonical pathways, e.g., by applying DNA-PKcs or ATM inhibitors, most likely hampers DSB repair in both normal and tumor cells. This effect leads to a limited therapeutic window due to normal-tissue toxicity. By contrast, targeting DSB repair stimulated by cytoplasmic signaling cascades, known as indirect targeting of DSB repair, is advantageous and seems to be a more tumor-specific approach for radiosensitization. However, the issue of tumor heterogeneity can potentially challenge the efficacy of this approach. This problem can be caused by functional crosstalk between different cytoplasmic signaling pathways or, alternatively, by a compensatory activation loop following the long-term inhibition of a target/pathway. This challenge mainly arises in tumors that harbor the overexpression and/or mutation of several oncogenes. Thus, to choose the appropriate target(s), it is necessary to identify the genetic background of tumors and develop biomarkers to predict the targetability of the components of a pathway that regulates DSB repair. This approach will help to select patients who will potentially benefit from the combination of radiotherapy with specific molecular targeting strategies for the indirect targeting of DSB repair.

## Figures and Tables

**Table 1:** Phase III clinical trials of EGFR monoclonal antibody cetuximab and early phase clinical trials of the EGFR tyrosine kinase inhibitor (TKI) erlotinib in combination with radiotherapy or chemoradiotherapy.

Target/Drug	Combination	Tumor Type	Outcome	Reference
EGFR/Cetuximab	RT	HNSCC	Improved OS	[46,47]
	CRT	NSCLC/Stage III	No improved OS	[53]
	CRT	Esophageal carcinoma	Reduced OS	[54]
	CRT	HNSCC	No improved OS	[55]
	RT vs. CRT	HPV-positive oropharyngeal carcinoma	Lower PFS after cetuximab + RT compared to CRT	[56]
EGFR/Erlotinib	RT	NSCLC	OS 62.5% (3 years)	[57]
	RT	Advanced or metastatic NSCLC	OS 30% (3 years)	[58]
	SBRT	NSCLC	PFS and OS greater than historical values	[59]
	CRT	NSCLC	Effective maintenance therapy PFS 63.5%	[60]
	Bavacizumab + CRT	HNSCC	OS 71% and PFS 82% (3 years)	[61]
	CRT	GM	No improvement in OS and PFS	[62]

RT: radiotherapy; HNSCC: head and neck squamous cell carcinoma; OS: overall survival; CRT: chemoradiotherapy; NSCLC: non-small cell lung cancer; PFS: progression-free survival; SBRT: stereotactic body radiation therapy; GM: glioblastoma multiforme; HPV: human papilloma virus.

**Table 2:** Clinical trials of the AKT antagonists in combination with radiotherapy or chemoradiotherapy.

Target/Drug	Combination	Tumor Type	Outcome	Reference
AKT/Nelfinavir	CRT/Phase II	Pancreatic cancer	Acceptable toxicity and promising activity	[160]
	RT/Phase I	Rectal cancer	Well-tolerated and good tumor regression	[161]
	CRT/Phase I	Rectal cancer	Nelfinavir 750 mg recommended phase II	[162]
	CRT/Phase I	NSCLC	Acceptable toxicity and promising activity	[163]
	CRT/Phase I	Pancreatic cancer	Acceptable toxicity and promising activity	[164]
AKT/Perifosine	RT/Phase I	NSCLC, prostate, esophageal, colon, and bladder cancer	Recommended phase II, 150 mg/day, started one week prior to RT	[158]
